# A. D. 1900

**Published:** 1900-01

**Authors:** 


**A Monthly Review of Medicine and Surgery.**

*EDITOR:*

WILLIAM WARREN POTTER, M. D.

All communications, whether of a literary or business nature, books for review and exchanges should be addressed to the editor: 284 FRANKLIN STREET, BUFFALO, N. Y.

VOL. XXXIX.—LV. JANUARY, 1900. NO. 6.

### A. D. 1900.

THE beginning of a new year is always a subject for interesting comment by individuals as well as newspapers and the periodical press. It is a time for thoughtful reflection, for a considerate review of the past, a hopeful contemplation of the future— and for good resolutions seldom wholly kept. This is generally the routine channel in which the thoughts of the average person flow.

What shall be said of such a period when it also marks the commencement of a new century? Is it not a time for nations to pause and consider what each is doing, and intends to do, for the betterment of mankind everywhere throughout the universe? Is it not a time for the professions to ask themselves what they can do to advance the interests of morality, restrain crime, and improve health?

It is not our purpose at present to discuss questions of ethics, religion, law or medicine in detail; or, indeed, to do more than suggest that the profession of medicine, proud of what it has already accomplished shall put its shoulder to the wheel, and with renewed vigor, conscious of its own prowess, push forward as one man with a mighty stride toward the ideals that its hallowed mission has created for itself.

It would be interesting, at this time, to review the progress of medicine during the 19th century, that inspiration or encouragement might be obtained for the future labors that await methodical and industrious effort. But this is not feasible here, nor, indeed, is it necessary; for every intelligent physician is more or less familiar with the facts.

We have in the JOURNAL within the past year or two presented a history of medicine in Erie County for the century just ended, in which it appears that physicians of Buffalo and its vicinity have been active participants in the work accomplished, contributing a liberal share to the general stock of medical knowledge upon continuously advancing lines.

It is more than probable that the principal work of the future will lie in the direction of the prevention of disease, and for any benefit to accrue from researches of this nature the public must look to legitimate medicine. Only thoroughly educated, well equipped and properly disciplined physicians are competent for this task. No others have ever successfully undertaken it and none ever will.

As indicating what is being done to increase longevity and prevent disease we may cite the records of the health department of Buffalo for the past ten years.

In 1890, population, 255,664, the ratio of deaths for every thousand was 19.32. The deaths from the principal infectious diseases were typhoid fever, 105; scarlet fever, 21; consumption, 498; pertussis, 16; measles, 21; cholera infantum, 348; diphtheria, 111.

In 1894, the population was estimated at 315,000, and it was a year of high death-rate from typhoid, because of the contamination of the city's water supply, a defect, however, that was speedily detected and swiftly remedied. In this year the deaths for every thousand of population were 16.76; from typhoid fever, 185; scarlet fever, 85; consumption, 500; pertussis, 73; measles, 11; cholera infantum, 417; diphtheria, 215.

Five years later—namely, in 1899, the figures were as follows: Population conservatively estimated at 370,000 (probably more nearly exact figures would be 400,000); deaths from typhoid fever, 98; scarlet fever, 31; consumption, 455; measles (epidemic), 52 cholera infantum, 187; diphtheria, 81.

The statistics here given are furnished by the registrar of vital statistics and are official. The last half of the month of December, 1899, was estimated—otherwise they are exact.

Here, then, is a steady decline in the deaths per thousand of population, notwithstanding a rapid increase in the growth of the city; besides, there has been a steady decline in the deaths from infectious or preventable diseases, the showing being particularly marked in cholera infantum and diphtheria. The first indicates the result of special attention to the city's milk supply by the health commissioner, and the second may be fairly attributed to the employment of antitoxin.

Can anything be more convincing as to the part played by scientific medicine in the prevention of disease? Does it not point directly to the proper relation of the state to medical education? Does it not indicate that the hope of the people rests with the best medical skill? Is it not a direct answer that puts to shame all believers in fads, isms, healers, and quacks?

The new century dawns under the influence of clearer skies; the year 1900 begins auspiciously, and we may look trustingly forward to a bright future that is steadily moving forward to crown us with daily mercies.

